# Sodium oligomannate combined with rivastigmine may improve cerebral blood flow and cognitive impairment following CAR-T cell therapy: A case report

**DOI:** 10.3389/fonc.2022.902301

**Published:** 2022-08-18

**Authors:** Yan-Li Wang, Yuan Zhang, Jun Xu

**Affiliations:** ^1^ Department of Neurology, Beijing Tiantan Hospital, Capital Medical University, Beijing, China; ^2^ China National Clinical Research Center for Neurological Diseases, Beijing Tiantan Hospital, Capital Medical University, Beijing, China

**Keywords:** sodium oligomannate, cognitive impairment, cerebral blood flow, CAR-T, rivastigmine

## Abstract

Chimeric antigen receptor-T (CAR-T) cell therapy is a breakthrough for B-cell hematological malignancies but is commonly associated with cytokine release syndrome and neurotoxicity and is occasionally complicated by neurological symptoms, such as cognitive disturbances. Currently, no effective treatments for CAR-T therapy-related cognitive impairment are available. Here, we present a 22-year-old patient with cognitive impairment who was treated with CAR-T cells as a salvage therapy for Burkitt lymphoma. One month after CAR-T cell infusion, he experienced memory loss that mainly manifested as forgetting recent-onset events. Two months of rehabilitation and hyperbaric oxygen therapy failed to provide clinical improvement. Subsequently, the patient improved with oral oxiracetam for 5 months. However, after 10 months of withdrawal, he showed significantly worse memory decline. Then, he began to take sodium oligomannate (22 February 2021). Follow-up testing at 6 and 12 months revealed maintenance of memory gains with sodium oligomannate alone or in combination with rivastigmine. Our case shows that CAR-T therapy may compromise cognitive function and that sodium oligomannate may have partial efficacy in restoring cognitive performance and activities of daily living. This may provide insights for further applications of sodium oligomannate for neurological symptoms, especially cognitive deficits following CAR-T cell therapy.

## Introduction

Burkitt lymphoma is a rare and highly aggressive B-cell neoplasm but is generally curable when treated with brief-duration, high-intensity chemotherapeutic regimens. However, some patients develop recurrence or fail to respond. Chimeric antigen receptor-T (CAR-T) cell therapy is an important innovative approach to treating hematological and other malignancies. However, cognitive impairment is an important symptom of neurotoxicity that is associated with a poor prognosis of CAR-T cell infusion (1). Currently, there is no effective intervention method, and this is the first case describing sodium oligomannate to control the progression of cognitive impairment after CAR-T cell therapy.

## Case report

A 22-year-old man was admitted to the Cognitive Disorders Department of our hospital in February 2021 complaining of gradual memory deterioration for more than 1 year.

The patient underwent CAR-T immunotherapy (specific treatment options not available) for Burkitt lymphoma in March 2019, and 1 month postoperatively, he first developed memory loss, with the main cognitive symptom being a failure to remember recent events. The patient suffered a grand mal seizure in April 2019, which was followed by a significant worsening of cognitive impairment, manifested by not recognizing familiar people, such as his parents, and not recognizing common objects, such as birds in paintings. MMSE and MoCA scores before treatment are unavailable. From June to August 2019, the patient was treated with rehabilitation and hyperbaric oxygen in our hospital with no significant improvement in cognitive symptoms. Since October 2019, the patient was administered oxiracetam 0.8 g orally twice daily for 5 months. The patient’s memory deterioration has improved, and he began to gradually recognize his parents and primary school and high school classmates and he can recall simple mobile phone passwords. However, the patient discontinued his medication due to the impact of COVID-19, and at month 15, he showed significantly worse memory decline, which primarily manifested as forgetting where to put things, emotional instability, and impaired daily living activities.

**Figure 1:**
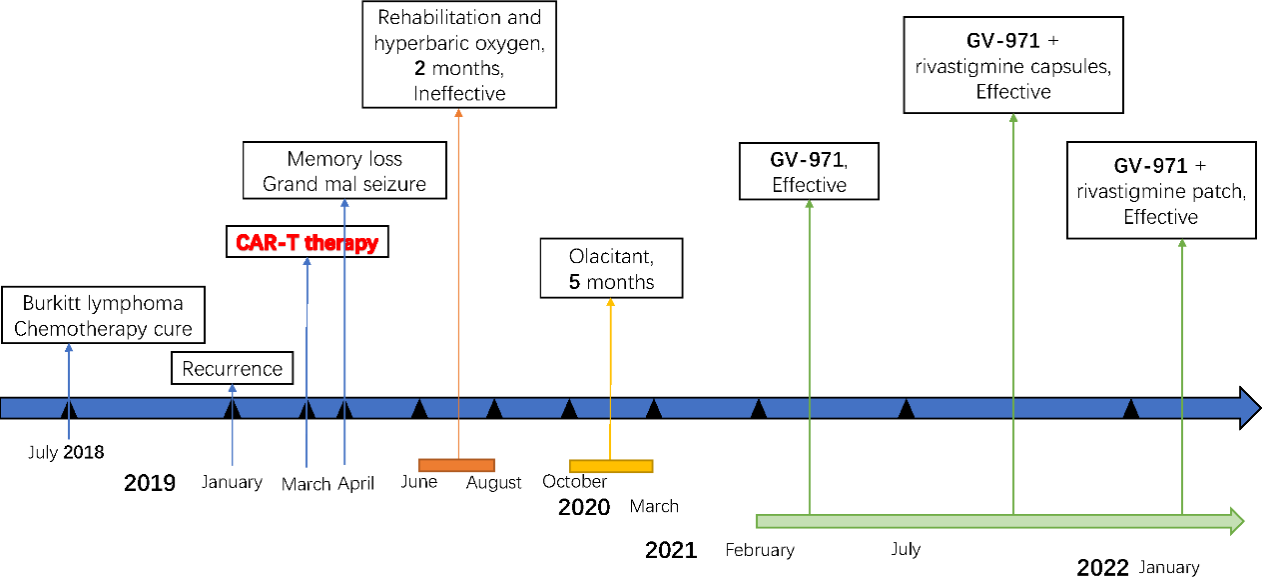
Medical history of the patient.

The study was approved by the Institutional Review Board of Beijing Tiantan Hospital of Capital Medical University (KY-2021-028-01). Informed consent was signed by the patient and his father. A timeline of the historical and current information is shown in **Figure 1**. Since February 2021, when the patient was seen in our outpatient clinic, the patient has been taking regular oral doses of sodium oligomannate (GV-971) 450 mg twice daily without any other medication. There were significant improvements in the overall condition of the patient, including improvements in the activities of daily living, more stable emotions, and increased MMSE and MoCA.

**Figure 2:**
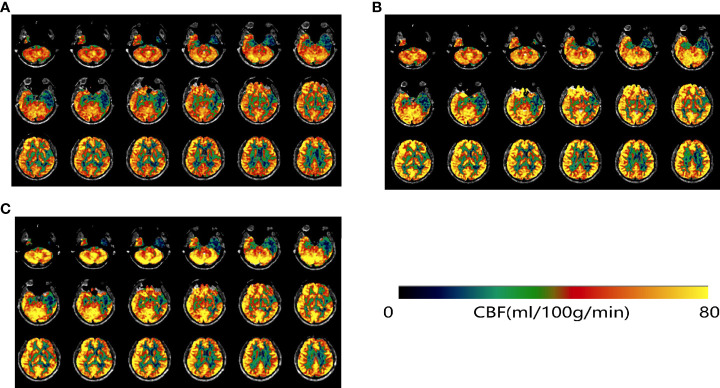
ASL-MRI on 9 February 2021 before GV-971 treatment **(A)**, 29 July 2021 **(B)**, and 18 January 2022 **(C)**. After treatment with GV-971 **(B, C)**, regions of low perfusion in green were visibly decreased.

To further improve his cognition, the patient has been co-administering rivastigmine hydrogen tartrate capsules 3 mg twice daily since August 2021; in January 2022, the regimen of 3 mg capsules twice daily was substituted with a 9.5-mg transdermal patch once daily. These were the only medications that the patient received. In comparison to sodium oligomannate alone, the patient was stable with no significant cognitive improvement. The results of the patient’s three neuropsychological examinations are shown in **Table 1**. The results for 14 cytokines and CAR-T cell copy numbers in peripheral blood samples from August 2021 are shown in **eTables 1**, **2**. **Figure 2** shows the comparison of cerebral perfusion findings with 3D arterial spin labeling (ASL) imaging before and after treatment with sodium oligomannate. Significantly, the hypoperfusion regions around the lesion improved following GV-971 treatment, and cerebral perfusion was stable during combination therapy with rivastigmine. Furthermore, **Table 2** shows cerebral blood flow (CBF) assessed with ASL in cognitive regions. The patient’s condition has improved steadily since the follow-up, with occasional fluctuations in symptoms. The patient’s cranial MRI in August 2021 showed that abnormal signals were present in the bilateral insula, hippocampus, amygdala, subcallosal gyrus, left temporal lobe, anterior cingulate, and splenium cingulate, which were not markedly changed from 6 months before. In addition, a neurological examination revealed poor memory and executive function, without other obvious abnormalities.

**Table 1:** Neurophysiological follow-up.

Follow-up		9 February 2021	29 July 2021	18 January 2022
		T0	T1	T2
Mini-Mental State Examination, MMSE		23	26	26
Montreal Cognitive Assessment, MoCA-Beijing		21	23	23
Hopkins Verbal Learning Test, HVLT	Short-Delayed Recall	0	6	0
	Long-Delayed Recall	0	1	0
Cog-12 Questionnaire Investigation, Cog-12		23	21	24
Hamilton Anxiety Scale, HAMA		4	5	5
Hamilton Depression Scale, HAMD		3	2	6
Neuropsychiatric Inventory Questionnaire, NPI		5	4	16
Activities of Daily Living, ADL-20		27	24	30
Pittsburgh Sleep Quality Index, PSQI		0	0	1

**Table 2:** Cerebral blood flow follow-up.

Cognition-related regions	Left lobe			Right lobe		
(mL/100g/min)	T0	T1	T2	T0	T1	T2
Hippocampus	28.59	26.30	37.21	29.83	36.39	37.03
Posterior cingulate	53.60	61.54	62.65	56.01	64.23	62.82
Parahippocampal gyrus	31.59	32.37	30.05	35.13	45.92	41.12
Thalamus	41.07	47.78	39.16	51.00	51.00	48.96
Amygdala	22.56	23.74	39.00	26.56	31.85	36.50
Precuneus	50.24	55.94	53.00	55.34	59.04	58.36
Middle temporal gyrus	47.01	55.85	55.66	56.21	64.21	75.08

## Discussion

After choosing to stop taking oxiracetam for 10 months, his MMSE score was 23 and his MoCA score was 21. After 6 months on sodium oligomannate, the patient improved significantly, with MMSE scores ranging from 23 to 26 and MoCA scores ranging from 21 to 23. The combination of sodium oligomannate with rivastigmine did not show significant cognitive improvement, but the effects were stable. Currently, the patient has a normal digital memory, can basically take care of himself, can use computers and other tools, and can interact with people around him in a normal way.

CAR-T cell therapy as a treatment for Burkitt lymphoma is a nascent, burgeoning field. Consistent with our observations, a previous study also indicated cognitive difficulties following CAR-T cell therapy (2). The specific mechanism may involve several factors, such as cytokine release syndrome (CRS) and immune effector cell-associated neurotoxicity syndrome (ICANS) (3). CRS typically occurs in the first week after CAR T-cell infusion and ICANS in the second week after infusion, usually following the peak of CRS severity and lasting 5 to 10 days (4). Mild ICANS (grades 1 and 2) is characteristic of aphasia, confusion, and impaired fine motor skills, and seizures, cerebral edema, and coma can manifest in severe cases (grades 3 and 4) (4). Both symptomatic treatment and supportive care are recommended to treat mild ICANS, whereas corticosteroids are the primary treatment strategy for severe ICANS (5, 6). Earlier administration of steroids could prevent severe effects without reducing CAR-T therapy efficacy (7). The severity of CRS is strongly correlated with the severity of ICANS, and approaches to CRS mitigation may subsequently reduce the risk of ICANS (8). Although tocilizumab has no effect on most ICANS, it is highly effective in the management of CRS (5, 9).

Although associations of early neurotoxicity with cognitive decline are significant, a large-scale cohort of patients receiving CAR-T cell therapy is needed to detect a correlation between acute neurotoxicity and long-term cognitive difficulties (2). There is also a study that found no cognitive toxicity of CAR-T cell treatment (10). The prevalence of cognitive difficulty following CAR-T cell therapy is higher among white individuals than among non-white patients, and a possible explanation for the discrepancies may arise from cultural differences (2).

A phase 3 clinical trial of sodium oligomannate demonstrated that GV-971 could effectively improve cognitive deficits in mild-to-moderate Alzheimer’s dementia (11).

Sodium oligomannate could reduce the concentration of phenylalanine and alleviate Th1-related neuroinflammation and cognitive decline by effectively modulating the gut microbiota (12). It may play an important role in preserving dendritic spines and synaptic plasticity (13). In addition, it can also directly inhibit the formation of Aβ fibrils and decrease Aβ deposition in the brain (14, 15). There are no studies that have evaluated the relationship between GV-971 and CBF, and the mechanism of GV-971 in cerebrovascular disease has not been explored. Patients with encephalitis and Alzheimer’s disease may exhibit regional CBF abnormalities, and CBF is a sensitive biomarker for the assessment of neuroinflammation and drug efficacy (16). This case strongly suggested that GV-971 might improve cognitive function by increasing cerebral blood flow.

## Conclusion

These results indicated that sodium oligomannate exerts its protective effect on cognitive improvement following CAR-T cell therapy through the inhibition of neuroinflammation and an increase in cerebral perfusion. Importantly, a larger cohort is needed to verify the results.

## Data availability statement

The original contributions presented in the study are included in the article/**Supplementary Material**. Further inquiries can be directed to the corresponding author.

## Ethics statement

This study was reviewed and approved by Institutional Review Board of Beijing Tiantan Hospital of Capital Medical University (KY-2021-028-01). The patients/participants provided their written informed consent to participate in this study.

## Author contributions

All authors have made a substantial contribution to the data collection and the drafting of the manuscript and reviewed and accepted the contents of the manuscript prior to its submission.

## Funding

This study was supported by the National Natural Science Foundation (Grant Numbers 82071187, 81870821, 81471215), The National Key Research and Development Program of China (2021YFC2500103), Beijing Youth Talent Team Support Program (2018000021223TD08), National Key Research and Development Program (2019YFC01209028), and Beijing Natural Science Foundation grant (L182055).

## Conflict of interest

The authors declare that the research was conducted in the absence of any commercial or financial relationships that could be construed as a potential conflict of interest.

## Publisher’s note

All claims expressed in this article are solely those of the authors and do not necessarily represent those of their affiliated organizations, or those of the publisher, the editors and the reviewers. Any product that may be evaluated in this article, or claim that may be made by its manufacturer, is not guaranteed or endorsed by the publisher.

## References

[1] Lee EQ . Neurologic complications of cancer therapies. Curr Neurol Neurosci Rep (2021) 21:66. doi: 10.1007/s11910-021-01151-w 34817688

[2] Ruark J Mullane E Cleary N Cordeiro A Bezerra ED Wu V . Patient-reported neuropsychiatric outcomes of long-term survivors after chimeric antigen receptor T cell therapy. Biol Blood Marrow Transplant J Am Soc Blood Marrow Transplant (2020) 26:34–43. doi: 10.1016/j.bbmt.2019.09.037 PMC6951812 31605820

[3] Brudno JN Kochenderfer JN . Recent advances in CAR T-cell toxicity: Mechanisms, manifestations and management. Blood Rev (2019) 34:45–55. doi: 10.1016/j.blre.2018.11.002 30528964 PMC6628697

[4] Morris EC Neelapu SS Giavridis T Sadelain M . Cytokine release syndrome and associated neurotoxicity in cancer immunotherapy. Nat Rev Immunol (2022) 22:85–96. doi: 10.1038/s41577-021-00547-6 34002066 PMC8127450

[5] Neelapu SS Tummala S Kebriaei P Wierda W Gutierrez C Locke FL . Chimeric antigen receptor T-cell therapy - assessment and management of toxicities. Nat Rev Clin Oncol (2018) 15:47–62. doi: 10.1038/nrclinonc.2017.148 28925994 PMC6733403

[6] Strati P Ahmed S Furqan F Fayad LE Lee HJ Iyer SP . Prognostic impact of corticosteroids on efficacy of chimeric antigen receptor T-cell therapy in large b-cell lymphoma. BLOOD (2021) 137:3272–6. doi: 10.1182/blood.2020008865 PMC8351896 33534891

[7] Shah NN Johnson BD Fenske TS Raj RV Hari P . Intrathecal chemotherapy for management of steroid-refractory CAR T-cell-associated neurotoxicity syndrome. Blood Adv (2020) 4:2119–22. doi: 10.1182/bloodadvances.2020001626 PMC7252557 32407473

[8] Freyer CW Porter DL . Cytokine release syndrome and neurotoxicity following CAR T-cell therapy for hematologic malignancies. J Allergy Clin Immunol (2020) 146:940–8. doi: 10.1016/j.jaci.2020.07.025 32771558

[9] Maus MV Alexander S Bishop MR Brudno JN Callahan C Davila ML . Society for Immunotherapy of Cancer (SITC) clinical practice guideline on immune effector cell-related adverse events. J Immunother Cancer (2020) 8(2):e001511. doi: 10.1136/jitc-2020-001511 33335028 PMC7745688

[10] Maillet D Belin C Moroni C Cuzzubbo S Ursu R Sirven-Villaros L . Evaluation of mid-term (6-12 months) neurotoxicity in b-cell lymphoma patients treated with CAR T cells: A prospective cohort study. Neuro-oncology (2021) 23:1569–75. doi: 10.1093/neuonc/noab077 PMC8408887 33822183

[11] Xiao S Chan P Wang T Hong Z Wang S Kuang W . A 36-week multicenter, randomized, double-blind, placebo-controlled, parallel-group, phase 3 clinical trial of sodium oligomannate for mild-to-moderate alzheimer's dementia. Alzheimer's Res Ther (2021) 13:62. doi: 10.1186/s13195-021-00795-7 33731209 PMC7967962

[12] Wang X Sun G Feng T Zhang J Huang X Wang T . Sodium oligomannate therapeutically remodels gut microbiota and suppresses gut bacterial amino acids-shaped neuroinflammation to inhibit alzheimer's disease progression. Cell Res (2019) 29:787–803. doi: 10.1038/s41422-019-0216-x 31488882 PMC6796854

[13] Ettcheto M Busquets O Cano A Sánchez-Lopez E Manzine PR Espinosa-Jimenez T . Pharmacological strategies to improve dendritic spines in alzheimer's disease. J Alzheimer's Dis JAD (2021) 82:S91–s107. doi: 10.3233/JAD-201106 33325386 PMC9853464

[14] Fan Y Hu J Li J Yang Z Xin X Wang J . Effect of acidic oligosaccharide sugar chain on scopolamine-induced memory impairment in rats and its related mechanisms. Neurosci Lett (2005) 374:222–6. doi: 10.1016/j.neulet.2004.10.063 15663967

[15] Jiang RW Du XG Zhang X Wang X Hu DY Meng T . Synthesis and bioassay of β-(1,4)-D-mannans as potential agents against alzheimer's disease. Acta Pharmacol Sin (2013) 34:1585–91. doi: 10.1038/aps.2013.104 PMC4002563 24241344

[16] Miao A Liu Q Li Z Liu W Wang L Ge J . Altered cerebral blood flow in patients with anti-NMDAR encephalitis. J Neurol (2020) 267:1760–73. doi: 10.1007/s00415-020-09747-x 32130498

