# Supplementary material for: Sodium oligomannate combined with rivastigmine may improve cerebral blood flow and cognitive impairment following CAR-T cell therapy: A case report
Source: Front Oncol. 2022 Aug 18;12:902301. doi: 10.3389/fonc.2022.902301 (PMC9433646; doi:10.3389/fonc.2022.902301)
Supplement: Supplementary file 1 [file DataSheet_1.pdf]

eTable 1. The B lymphocyte recovery test in peripheral blood of post-CAR-T therapy

August 23, 2021	Results	Reference range
lymphocyte ratio	37.18%	20-40%
T lymphocyte ratio	61.99%	50-84%
CD3+CD4+	22.60%	20-50%
CD3+CD8+	31.20%	15-45%
CD4/CD8 ratio	1：1.38	0.70-2.7
B cell ratio	12.52%	5-18%
NK cell ratio	24.23%	7-40%
NKT cell ratio	2.56%	0.78-11.71%
cCD79a+ cell ratio	12.3%	-
CD20+ cell ratio	11.94%	-
CD22+ cell ratio	12.07%	-

eTable 2 Levels of 14 inflammatory cytokines

August 23, 2021	Results (pg/mL)	Reference range
IL-1β	1.53	0-3.4
IL-2	0.00	0-6.64
IL-4	0.53	0-4.19
IL-5	0.55	0-4.15
IL-6	9.78	0-11.09
IL-8	36.29	0-15.71
IL-10	1.56	0-4.5
IL-12p70	0.31	0-10.18
IL-17A	1.65	0-4.74
IL-17F	0.95	0-4.66
IL-22	2.08	0-3.64
TNF-α	3.72	0-4.5
TNF-β	0.00	0-2.54
γ-IFN	0.73	0-4.43
